# Genetic and clinical study of *PARK7* in Japanese Parkinson's disease

**DOI:** 10.1016/j.heliyon.2024.e35271

**Published:** 2024-07-26

**Authors:** Mayu Ishiguro, Manabu Funayama, Taku Hatano, Hiroshi Nishida, Yuko Wada, Kazuyuki Noda, Masahiko Tomiyama, Hiroyo Yoshino, Yuanzhe Li, Stephanie Ong, Ettore Cioffi, Kenya Nishioka, Nobutaka Hattori

**Affiliations:** a Department of Neurology, Faculty of Medicine, Juntendo University, Tokyo, Japan; b Research Institute for Diseases of Old Age, Graduate School of Medicine, Juntendo University, Tokyo, Japan; c International Collaborative Research Administration, Juntendo University, Tokyo, Japan; d Department of Neurology, Gifu Prefectural General Medical Center, Gifu, Japan; e Department of Neurology, Rakuwa-kai Otowa Hospital, Kyoto, Japan; f Department of Neurology, Juntendo University Shizuoka Hospital, Shizuoka, Japan; g Department of Neurology, Institute of Brain Science, Hirosaki University Graduate School of Medicine, Hirosaki, Japan; h Department of Diagnosis, Prevention and Treatment of Dementia, Graduate School of Medicine, Juntendo University, Tokyo, Japan; i Department of Neurology, Juntendo Tokyo Koto Geriatric Medical Center, Tokyo, Japan; j Neurodegenerative Disorders Collaborative Laboratory, RIKEN Center for Brain Science, Saitama, Japan

**Keywords:** Familial Parkinson's disease, DJ-1, Genetics

## Abstract

**Background:**

Biallelic variants in *PARK7*, which encodes protein-nucleic acid deglycase DJ-1, can cause early-onset Parkinson's disease (PD). Although many patients with *PARK7* variants have been identified from European and Middle Eastern ethnic groups, there have been no reports in the Japanese population.

**Objectives:**

To determine the prevalence and clinical features of patients with PD harboring *PARK7* variants in Japan.

**Methods:**

We performed a molecular genetic analysis of PD patients with *PARK7* variants identified using comprehensive panel sequencing, to explore the details of variants. Moreover, clinical neurological features were investigated, including neuroimaging analyses. This study followed STROBE guidelines.

**Results:**

Four patients with biallelic rare variants of *PARK7* were identified in the cohort. All four patients presented with levodopa-responsive parkinsonism, with an age at onset in the early 30s. Furthermore, two of the four patients had psychiatric complications. Dopamine transporter imaging revealed nigrostriatal pathway dysfunction.

**Conclusions:**

To our knowledge, this is the first report of Japanese patients with *PARK7* variants. We identified a relatively low frequency of *PARK7* variants in patients in Japan. As opposed to typical patients with sporadic PD, the identified patients developed the disease in their 30s and presented with a variety of non-motor symptoms and complications. Further studies are needed to identify the clinical features related to *PARK7* variants in Japanese patients with PD, and to analyze the pathophysiology of how the variants identified in the present study might affect DJ-1 function.

## Highlights

• Four patients with biallelic rare variants of *PARK7* were identified in the Japanese population.
• The prevalence in early-onset PD was very rare; this frequency is consistent with previous reports.
• PD patients with *PARK7* mutations were revealed to develop at a early age and to have a clinically diverse presentation.

## 1 Introduction

Parkinson's disease (PD) is the second most common neurodegenerative disease after Alzheimer's disease, affecting more than 1 % of individuals over 60 years of age [1,2]. The most prominent clinical features of PD are motor symptoms such as bradykinesia, rigidity, and resting tremor, which have an excellent response to levodopa therapy. Although the motor symptoms of PD are caused by the degeneration of dopaminergic neurons in the substantia nigra, patients also present with various non-motor symptoms that are caused by neurodegeneration of the non-dopaminergic system. The etiology of PD remains unclear and there are currently no curative therapies; however, it is anticipated that the identification of disease-related genes from patients with familial PD will contribute to breakthroughs in the study of PD. More than 30 different causative genes for familial PD have been reported, including dominant types of PD caused by variants in synuclein alpha (*SNCA*) and leucine rich repeat kinase 2 (*LRRK2*), and recessive types caused by variants in parkin RBR E3 ubiquitin protein ligase (*PRKN*), PTEN induced kinase 1 (*PINK1*), and Parkinsonism associated deglycase (*PARK7*, or *DJ-1*) [2].

*DJ-1* was initially discovered as an oncogene; however, in 2003, Bonifati and colleagues identified two biallelic variants from two separate consanguineous families with early-onset PD (MIM#606324), thus identifying *DJ-1* as a recessive causative gene for familial PD [3,4]. *PARK7* is located on chromosome 1p36 and consists of seven exons encoding 189 amino acids. The gene product, DJ-1, has a molecular weight of 22 kDa, is highly conserved across species, and is widely expressed in various tissues, especially the brain and testis [5]. Since the original report of *PARK7* as a PD-related gene, many validation studies have been conducted in diverse populations; however, the prevalence of pathogenic *PARK7* variants in PD patients appears to be relatively low [6]. To better understand the function of DJ-1 and the detailed genetic and clinical features associated with *PARK7* biallelic variants, it is essential to study the etiology of DJ-1 and early-onset PD by accumulating as many patients as possible. Here, we investigated the characteristics of *PARK7* variants in Japanese patients with PD, and explored the associated clinical features. We identified four patients from independent families with biallelic *PARK7* variants in our Japanese PD cohort, and analyzed their clinical courses. Our findings provide a further understanding of the impact of *PARK7* variants on clinical features of PD.

## 2 Subjects and methods

### 2.1 Participants

**Fig. 1:**
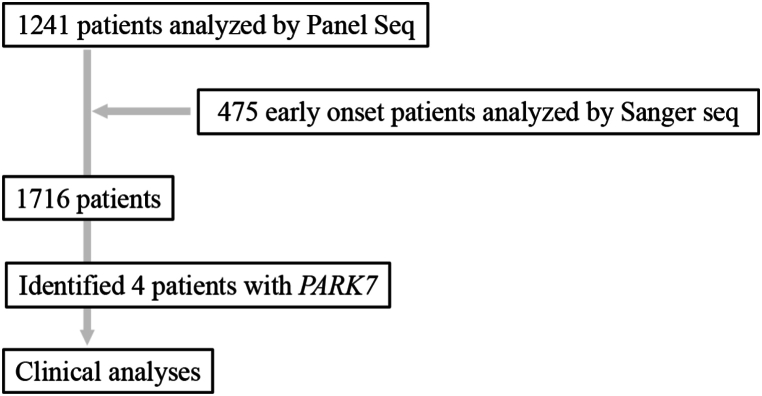
Study flow chart. In the study, 1716 patients were analyzed using panel sequencing (seq) or Sanger seq. Pathological variants of *PARK7* were identified in four patients.

This study was approved by the ethics committee of the Juntendo University Faculty of Medicine (approval number M08-0477). All participants gave informed written consent before participation, and all patients met the standard diagnostic criteria of PD [1,7]. Medical records were used to obtain clinical data such as clinical course; symptoms; and the results of neuroimaging such as brain magnetic resonance imaging (MRI), ^123^I-metaiodobenzylguanidine (MIBG) myocardial scintigraphy, dopamine transporter single-photon emission computed tomography (DAT-SPECT), ^99m^Tc-ethyl cysteinate dimer SPECT (^99m^Tc-ECD-SPECT), and *N*-isopropyl-*p*-^123^I-iodoamphetamine SPECT (^123^I-IMP-SPECT). A total of 1716 Japanese patients with PD participated in the study. Patients in whom rare variants in PD-related genes other than *PARK7* were identified before the start of this study were excluded. The cohort comprised 1241 patients with PD who underwent panel sequencing (described in section 2.2) from June 2016 to March 2023, and 475 patients who did not undergo panel sequencing as an additional analysis and who were under 50 years of age at onset. Patients considered to have developed PD due to *PARK7* variants were subjected to neurological analyses. A study flow chart is shown in Fig. 1.

### 2.2 Mutation screening

We extracted genomic DNA from the peripheral blood of patients using the QIAamp DNA Blood Maxi kit (Qiagen, Venlo, Netherlands). Patients were screened for genes related to familial PD or dementia (panel ID: IAD103177_182) using high-throughput next-generation sequencing with an Ion Torrent System (Thermo Fisher Scientific, Waltham, MA, USA). The methods of our targeted gene panel screening have been described previously [8]. Sanger sequencing was performed to validate non-synonymous variants detected by panel sequencing. Multiplex Ligation-Dependent Probe Amplification (MLPA) was used for copy number variant analysis using the SALSA MLPA® P051 Parkinson probe mix (MRC Holland, Amsterdam, Netherlands). Raw data for gene dosage quantification were collected on a SeqStudio sequencer (Thermo Fisher Scientific) and analyzed with Coffalyser software (MRC Holland). Probe ratios below 0.7 or above 1.3 were defined by the Coffalyser software as indicating a heterozygous deletion or a heterozygous duplication, respectively. Haplotype analysis was performed by fragment analysis and the genotyping of microsatellites located in flanking *PARK7* using fluorescently labeled primers on a SeqStudio sequencer, and by the genotyping of single nucleotide variants using Sanger sequencing. Haplotype phases were determined by comparison with haplotypes from the parents of Patient D–II–2. The primer sequence information (for the Sanger sequencing and haplotype analysis) is available upon request.

### 2.3 Quantitative reverse transcription polymerase chain reaction (qPCR)

qPCR was performed to quantify *PARK7* mRNA. Total RNA was collected from peripheral blood using the PAXgene RNA system (BD, Franklin Lakes, NJ, USA) according to the manufacturer's protocol. cDNA was synthesized from total RNA using the ReverTra Ace qPCR RT Master Mix (TaKaRa Bio, Tsu, Japan). qPCR was performed on the QuantStudio 7 Flex Real-Time PCR System (Thermo Fisher Scientific) using PowerUp SYBR Green Master Mix (Thermo Fisher Scientific) and *PARK7-*specific qPCR primers. Primer3Plus was used for primer design [9], and at least one primer was designed to be located at the exon–exon junction and amplify cDNA only. Two targets (exons 2–3 and exon 7) were set in *PARK7*. The qPCR experiments were performed in triplicate and repeated three times. Relative expression levels were calculated using *ACTB* as the reference, and were compared statistically by one-way analysis of variance and Tukey's post hoc test using JMP17 (JMP, Cary, NC, USA). The qPCR primer sequence information is available upon request.

### 2.4 Bioinformatic analysis

For this bioinformatic analysis, NM_007262 was used as the reference sequence. The pathogenicity of each missense variant was estimated using Rare Exome Variant Ensemble Learner (REVEL) [10]. Furthermore, the Single Nucleotide Polymorphism Database (dbSNP) [11] was used to search for the allele frequencies of previously reported variants.

## 3 Results

### 3.1 Age demographics of the participants

Of the 1716 patients with PD who were enrolled in the present study, 688 had familial PD (with the same phenotype identified in the same family) and 1028 had sporadic PD. The detailed age characteristics of the participants are described in Table 1. The mean age at onset was 14.4 years earlier in sporadic PD than in familial PD; this difference may have been caused by a sample selection bias because most of the early-onset PD cases who were analyzed additionally using Sanger sequencing had sporadic PD (Fig. 1).

**Table 1:** Age characteristics of individuals.

	Number of patients (male: female)	Age at sampling, y [mean ± SD (range)]	Age at onset, y [mean ± SD (range)]	Disease duration, y [mean ± SD (range)]
Total patients	1716 (935 : 781)	53.9 ± 13.6 (12–94)	46.4 ± 13.6 (7–88)	7.5 ± 7.5 (0–72)
Familial PD	688 (350 : 338)	62.2 ± 12.8 (17–94)	55.0 ± 13.9 (7–88)	7.2 ± 7.4 (0–52)
Sporadic PD	1028 (585 : 443)	48.3 ± 11.3 (12–88)	40.6 ± 9.7 (7–83)	7.7 ± 7.5 (0–72)

PD, Parkinson's disease; SD, standard deviation; y, years.

### 3.2 Frequency of PARK7 variants in Japanese patients with PD

In our cohort, we identified four patients with biallelic rare variants of *PARK7*. The frequencies were 0.23 % of the total cohort, 0.29 % (2/688) of patients with familial PD, and 0.19 % (2/1028) of patients with sporadic PD. The prevalence of variants in patients with PD with an age at onset <50 years was 0.34 % (4/1168). No *PARK7* variants were identified in patients with PD with an age at onset ≥50 years.

### 3.3 Identified biallelic PARK7 variants in PD patients

**Fig. 2:**
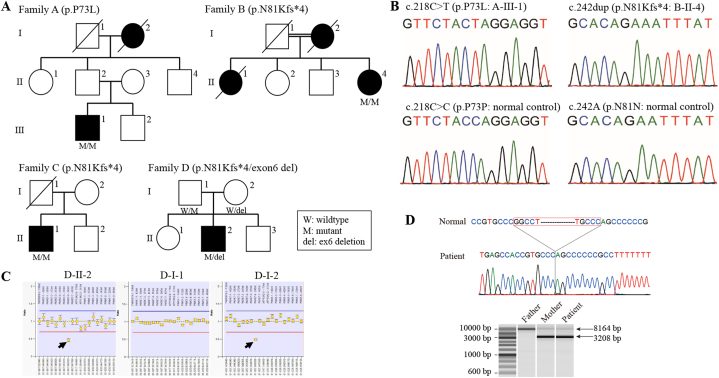
**Mutation analysis of*****PARK7*****.** A: Family tree of PD patients with biallelic variants of *PARK7*. B: Electropherograms of pathological single nucleotide variations detected in this study. C: Results of the MLPA of Family D. D: Sequencing electropherogram of the deletion breakpoint from Patient D–II–2.

**Fig. 3:**
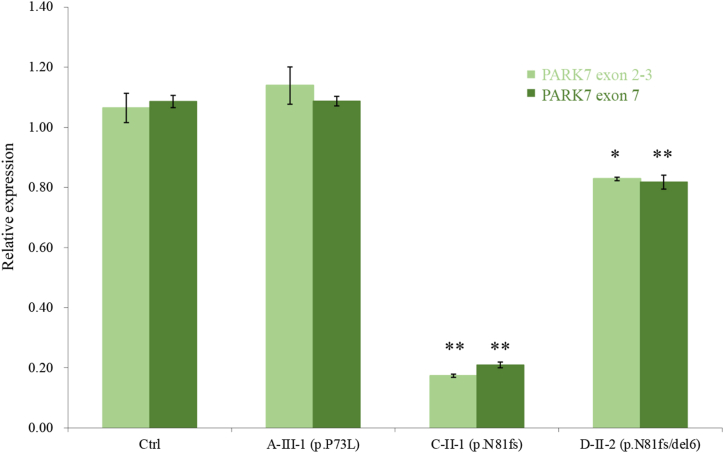
**Quantification of*****PARK7*****mRNA.** Relative *PARK7* mRNA expression, measured using qPCR. Two targets—exons 2–3 and exon 7—were set in *PARK7. PARK7* mRNA expression levels were compared between subjects without *PARK7* variants (ctrl) and those with the biallelic p.P73L variant (A–III–1), the biallelic p.N81Kfs*4 variant (C-II-1), or the compound heterozygous p.N81Kfs*4 variant and exon 6 deletion (D–II–2). *P < 0.05 vs. ctrl, **P < 0.001 vs. ctrl. Error bars represent standard errors. The bar chart was drawn using the third qPCR data. All three qPCR experiments showed similar trends.

Two rare variants and one exonic deletion were identified in four patients with PD from independent families: a homozygous c.218C > T (p.P73L) variant in one patient (A–III–1), a homozygous c.242dup (p.N81Kfs*4) variant in two patients (B-II-4 and C-II-1), and a compound heterozygous c.242dup (p.N81Kfs*4) variant and exon 6 deletion in one patient (D–II–2) (Fig. 2A–C). Further exploration of the exonic deletion breakpoint revealed that 4.9 kbp of hg38 chr1:7977016_7981953 was heterozygously deleted in Patient D–II–2 (Fig. 2D). When we compared the 300 bases forward of the deletion with the 300 bases at the deletion site, there was 86 % homology. Moreover, c.218C > T (p.P73L) was registered as rs367584305 and c.242dup (p.N81Kfs*4) was registered as rs1640403698; both were rare variants, especially rs1640403698, which has been detected in the Japanese population only. No other putative pathogenic variants in PD-related genes were identified via panel sequencing in these patients. In silico prediction by REVEL showed that c.218C > T (p.P73L) had a high REVEL score, of 0.837. In addition, c.242dup (p.N81Kfs*4) was predicted to produce a stop codon after four amino acids and cause nonsense-mediated mRNA decay. Indeed, the analysis of *PARK7* mRNA expression in Patients C-II-1 and D–II–2, from whom RNA was able to be obtained, showed a prominent and significant decrease compared with controls (without *PARK7* variants) (Fig. 3). Furthermore, c.242dup (p.N81Kfs*4) was identified in three of the four patients with *PARK7* variants in our cohort. Patients C-II-1 and D–II–2 may have shared a common founder with a maximum range of approximately 1.1 Mbp (including *PARK7*), whereas B-II-4 had less useful and inconclusive genetic marker information (Table 2). We also detected a c.310G > A (p.A104T) variant in one PD patient; however, although c.310G > A (p.A104T) has been reported as a PD-associated variant, its pathogenicity remains unknown because it was a monoallelic variant (data not shown) [12].

**Table 2:**
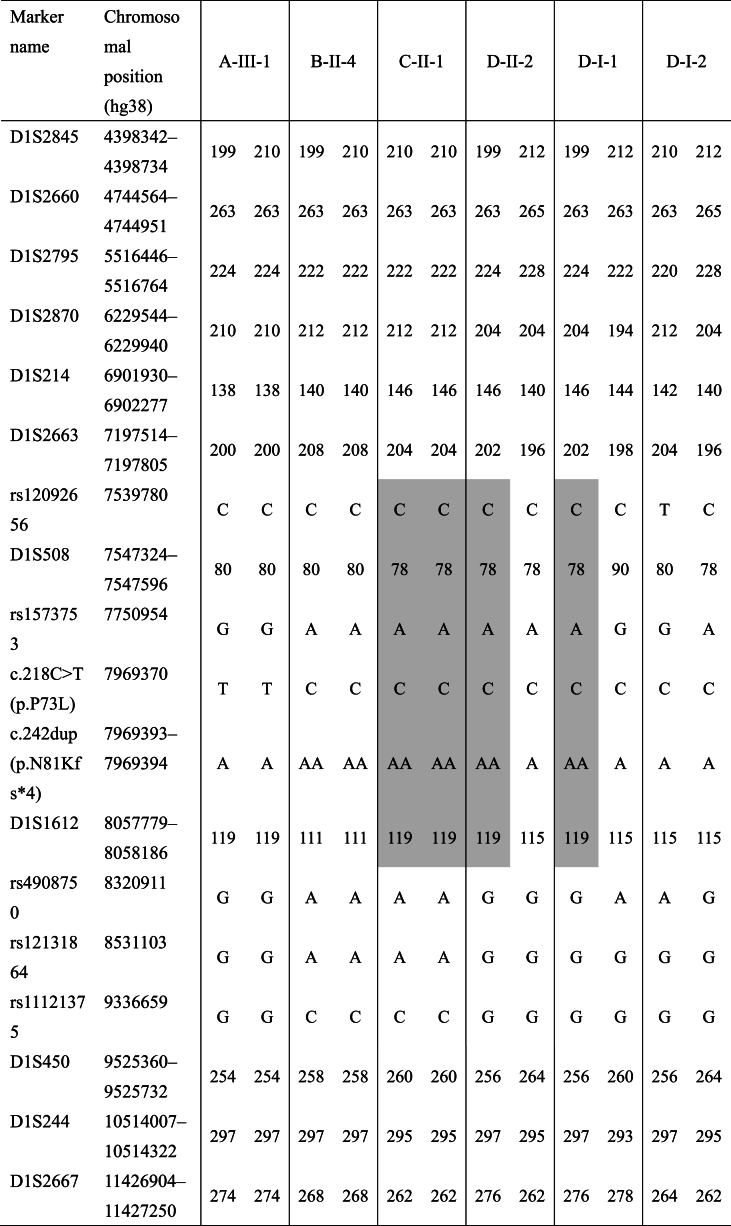
**Haplotype analysis of*****PARK7*****rare variant carriers.**

Estimated common haplotypes are indicated by gray backgrounds.

### 3.4 Clinical findings of patients with biallelic PARK7 variants

**Fig. 4:**
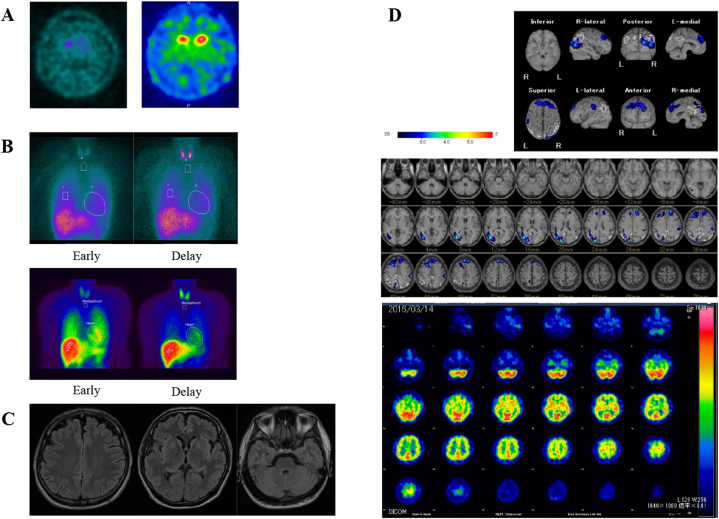
**Neuroimaging of patients with*****PARK7*****variants.** A: DAT-SPECT of Patients A–III–1 (left) and C-II-1 (right). B: ^123^I-MIBG myocardial scintigraphy of Patients A–III–1 (upper) and C-II-1 (lower). C: Brain MRI of Patient C-II-1. D: ^99m^Tc-ECD-SPECT of Patient A–III–1.

Patient A–III–1 was a 48-year-old man who had experienced panic attacks since his teens. At the age of 35 years, he presented gait disturbance and dysphonia and was diagnosed with PD. Aged 40 years, wearing-off and psychiatric symptoms such as hallucinations, auditory hallucinations, and delusions appeared. His psychiatric symptoms improved with the discontinuation of dopamine agonists. During the off-state, he experienced dysarthria with stuttering and difficulty walking. At the age of 43 years, he frequently overdosed on levodopa, and was diagnosed with dopamine dysregulation syndrome. When his levodopa medications increased, he had increased agitation and irritability. From 45 years of age, he has been repeatedly hospitalized for long periods in mental hospitals to control his agitation. At the age of 48 years, his activities of daily living classify him as Hoehn and Yahr stage III. He has not experienced any cognitive decline. His younger sister had intellectual disability from birth without any movement disorders. Interviews with the patient's parents revealed that the patient's grandmother may also have had PD. Furthermore, given that the family's relatives lived in a historically closed area, the possibility of an accidental consanguineous marriage cannot be ruled out. In terms of imaging, the patient's brain MRI was normal. DAT-SPECT showed a reduction of dopamine transporter uptake (Fig. 4A). His heart to mediastinum (H/M) ratio did not decrease in MIBG myocardial scintigraphy (Fig. 4B). ^99m^Tc-ECD-SPECT showed lower uptake in the right posterior lobe (Table 3 and Fig. 4D).

**Table 3:** Clinical features of patients with biallelic *PARK7* variants.

ID number	A–III–1	B-II-4	C-II-1	D–II–2
Sex	Male	Female	Male	Male
Age at examination (y)	47	69	36	39
Age at disease onset (y)	35	32	32	33
H–Y stage	3	5	2	3
Family history	+	+	–	–
Resting tremor	–	+	+	–
Bradykinesia	+	+	+	+
Rigidity	+	+	+	+
Postural instability	+	+	NA	+
Gait disturbance	+	+	NA	–
Levodopa responsive	+	+	+	+
Wearing off	+	+	NA	–
Levodopa-induced dyskinesia	+	–	NA	–
Levodopa-induced dystonia	–	–	NA	NA
Asymmetry at onset	–	NA	NA	+
Dystonia	–	+	+	–
Deep tendon reflex	–	–	NA	+
Plantar reflex	–	–	NA	–
Constipation	–	+	NA	–
Incontinence	NA	+	NA	–
Orthostatic hypotension	–	–	NA	–
Tachycardia	–	–	NA	NA
Insomnia	NA	+	NA	NA
RBD	+	–	NA	–
Cognitive decline	–	–	NA	+
Depression	–	–	NA	–
Anxiety	NA	–	NA	–
Delusion	+	–	NA	–
Hallucination	+	–	NA	–
Dysarthria	+	+	NA	NA
Neuropathy	–	+	NA	NA
Atrophy	–	–	NA	–
Cerebellar ataxia	–	–	NA	–
Brain MRI	Normal	Normal	Normal	Normal
MIBG myocardial scintigraphy	Normal	Normal	Decreased	Normal
Brain SPECT	Decreased in frontal lobe	Decreased in occipital lobe	NA	Decreased in occipital lobe
DAT-SPECT SBR	Decreased	NA	Decreased	Decreased

-, absent; +, present; DAT, dopamine transporter; H–Y, Hoehn and Yahr; ID, identification; MIBG, ^123^I-metaiodobenzylguanidine; MRI, magnetic resonance imaging; RBD, rapid eye movement behavior disorder; NA, not applicable; SBR, striatal binding ratio; SPECT, single-photon emission computed tomography.

Patient B-II-4 was a 69-year-old woman. She noticed gait disturbance, resting tremor, and bradykinesia at the age of 32 years. Her older sister developed PD in her 30s and passed away aged 45 years, and her unaffected parents had a consanguineous marriage. The patient was diagnosed with PD, and levodopa ameliorated her parkinsonism. She developed hallucinations and delusions in her 40s. Dystonia of both lower extremities appeared at 47 years of age. At the age of 58 years, she reported sensory disturbances with hyporeflexia of all limbs; a nerve conduction study revealed axonal neuropathy. She has been bedridden since the age of 60 years, without any cognitive decline. In terms of imaging, the patient's brain MRI was normal, and DAT-SPECT showed a reduction of dopamine transporter uptake. Her H/M ratio did not decrease in MIBG myocardial scintigraphy. ^123^I-IMP-SPECT showed lower uptake in the frontal lobe (Table 3).

Patient C-II-1 was a 36-year-old man who developed tremor at the age of 32 years. He had no family history, and demonstrated good levodopa responsiveness. He presented with cervical dystonia, which improved with oral levodopa and dopamine agonists. Aged 36 years, he was taking 250 mg levodopa and 9 mg rotigotine and was classified as Hoehn and Yahr stage II. His brain MRI was normal (Fig. 4C). DAT-SPECT showed a reduction of dopamine transporter uptake (Fig. 4A). His H/M ratio was decreased in MIBG myocardial scintigraphy (Table 3 and Fig. 4B).

Patient D–II–2 was a 39-year-old man. He developed a resting tremor in his right hand at the age of 32 years. Aged 34 years, he had a shuffling gait. He then developed spasticity, and at the age of 37 years, he was suspected to have hereditary spastic paraplegia. His spasticity worsened, and he required assistance to walk. Myoclonus of the upper extremities was observed. In a levodopa challenge test, he showed improvements in masked face and stiffness of the upper extremities, but his improvement rate was 23 % in part III of the Movement Disorder Society-Unified Parkinson's Disease Rating Scale. He had cognitive decline, with a Mini-Mental Scale Examination score of 14 points. In imaging findings, his brain MRI was normal. DAT-SPECT showed a reduction of dopamine transporter uptake. His H/M ratio did not decrease in MIBG myocardial scintigraphy. ^123^I-IMP-SPECT showed lower uptake in the frontal and temporal lobes (Table 3).

## 4 Discussion

In the present study, we screened a large Japanese PD population for patients with *PARK7* variants; we identified four patients with early-onset PD who had pathogenic biallelic variants. Of the Japanese PD patients with an age of onset <50 years, 0.34 % had biallelic *PARK7* variants; this frequency is consistent with previous reports [6]. However, it should be noted that our cohort excluded PD cases with previously identified pathological variants in PD-related genes other than *PARK7*, so the actual frequency was expected to be even lower. Notably, in three of the four PD patients, we detected a frameshift variant that resulted from the same type of single nucleotide insertion. Haplotype analysis suggested that at least two of the families with this variant shared a common founder. Moreover, database analysis revealed that this variant has been identified in the Japanese population only, suggesting that it may be a Japanese-specific variant. In addition, a single nucleotide substitution variant that was detected in the present study has been reported at very low allele frequencies in various ethnic groups. Furthermore, the encoded amino acids are highly conserved across species. The identified missense variants were determined as pathogenic by REVEL, suggesting that these biallelic variants are causative for PD development. In addition, one patient had a heterozygous deletion in exon 6, and a comparison of the 300 bases forward of the deletion with the 300 bases at the deletion site showed 86 % homology. This site is therefore considered likely to be susceptible to recombination. By contrast, c.310G > A (p.A104T), which has already been reported as pathogenic for PD, was detected heterozygously in one patient with PD and its pathogenicity is unclear [12].

Most previously reported patients with *PARK7* variants had PD onset in their 20s–30s, with rare cases of disease onset in infants and teenagers [[13], [14], [15], [16], [17]]. Although the reported clinical features have been variable, patients generally present with good levodopa responsiveness, dystonia, and dyskinesia [13]. In the present study, all patients shared a common onset of parkinsonism in their 30s. However, two patients (A–III–1 and D–II–2) had psychiatric symptoms, whereas two others (B-II-4 and C-II-1) with the same type of biallelic variants had dystonia. A range of complications are often reported in patients with *PARK7* variants, including dysphagia, dysarthria, peripheral neuropathy [16,18,19], pyramidal tract disorders, dysphonia, and muscle atrophy; however, it may be that the type of complications that develop depend on the variant of each patient [15,[18], [19], [20]]. Cases with p.E163K and p.Q45X variants are reported to be complicated with amyotrophic lateral sclerosis [21,22]. Interestingly, Patient B-II-4 had axonal peripheral neuropathy, and Patient D–II–2 had severe spasticity mimicking hereditary spastic paraplegia. In addition to amyotrophic lateral sclerosis complications, pyramidal tract signs were reportedly positive in three studies that mentioned pyramidal tract signs [[14], [15], [16],^18^,^19^,^23^]. Together, these findings suggest that *PARK7* variants might also be associated with motor neuron degeneration. In all of our cases, brain MRI showed no abnormalities. Similarly, most previously reported patients with biallelic *PARK7* variants reported have shown no abnormalities, although a few cases showed cortical or cerebellar atrophy, suggesting that *PARK7* variants do not substantially affect brain atrophy [16,18]. Striatal dopamine transporter binding was low in all three examined patients, and three of our four patients showed normal H/M ratio values. In our previous studies, H/M ratios were heterogeneous in patients with familial PD-associated genes [8,[24], [25], [26]]. *PARK7* variants may therefore lead to a loss of DJ-1 function, striatal neuronal loss, and typical symptoms of PD (i.e., with similar motor symptoms, age of onset, and responsiveness to levodopa therapy as typical PD). However, *PARK7* variants may also cause various non-motor symptoms, psychiatric symptoms, and neuromuscular diseases with different variants and disease durations.

The monomeric DJ-1 protein contains seven β-strands and nine α-helices in a three-layered structure, with α-helices on either side of the parallel β-sheet [5]. Previous studies have shown that DJ-1 forms dimers, which are made by interactions of the β-sheet β3 and the α-helices α1, α8, and α9. The H-bond between C-terminal residues Pro 184 and His126 is essential for dimer formation [27]. In the case of Patient D–II–2, the deletion of exon 6 suggests that the C-terminal structure of the DJ-1 protein remains intact, because exon 7 is expected to remain in-frame. However, the deletion of His126 encoded in exon 6 is expected to prevent dimer formation. Chen and colleagues have also reported that His126 is an essential amino acid for the protease activity of DJ-1 [28]. Together, these findings suggest that this amino acid is crucial for multiple functions of DJ-1, including dimer formation and protease activity. For the p.P73L variant, Pro 73 usually exists in close proximity to β4 in the β-sheet structure in the DJ-1 molecule. The amino acid substitution from proline to leucine is therefore expected to reduce the structural stability of DJ-1. The p.N81Kfs*4 variant led to significantly lower mRNA expression in the current study, suggesting that transcripts are rapidly degraded by nonsense-mediated mRNA decay, and that there is little or null DJ-1 protein.

The present study includes some limitations. First, the clinical data were evaluated as a cross-sectional study, without concern for time differences in each patient. Second, we did not perform any functional analyses using cell culture or model animals. Finally, because there is a high proportion of samples from cases of familial PD in our DNA bank, the prevalence reported in the present study might differ slightly from that of a general population of PD patients.

## 5 Conclusions

In conclusion, patients with *PARK7* variants were rare in the Japanese population. Patients presented in their 30s and showed various clinical symptoms alongside levodopa-responsive parkinsonism. Further studies are needed to elucidate the mechanisms by which *PARK7* variants may lead to PD.

## Ethics and consent statement

This study was reviewed and approved by the Research Ethics Committee of the Faculty of Medicine, Juntendo University, Tokyo, Japan with the approval number: M08-0477, dated August 1st, 2023. All patients or their proxies/legal guardians provided written informed consent to participate in the study and for the publication of their anonymized case details and images. Written consent was obtained from the patient and their parent or guardian in the case of minors aged 16 years and over, and from the parent or guardian in the case of patients under 16 years of age. For minors under 16 years of age with a certain level of understanding, although consent was obtained from the parent or guardian, efforts were made to explain the procedure to the patient and obtain their understanding. Consent was also obtained from the parent or guardian if it was difficult to obtain consent from a patient aged 16 years or older because the patient had a condition such as impaired consciousness, cognitive impairment, or intellectual disability, or because the patient had already died when samples collected in another research project were used in the present study.

## Funding

This work was supported by the 10.13039/501100001691Japan Society for the Promotion of Science (10.13039/501100001691JSPS) 10.13039/501100001691KAKENHI [Grant Numbers: 24K02372 for MF, 22K07542 for 10.13039/100007797HY, 21K07283 for YL, and 21H04820 for NH], the 10.13039/501100002241Japan Science and Technology Agency 10.13039/501100020963Moonshot R&D Program [Grant Number: JPMJMS2024-5 for NH], and the 10.13039/100009619Japan Agency for Medical Research and Development (10.13039/100009619AMED) [Grant Numbers: JP23bm1423015h0001 for MF and NH, JP23dm0207070 and JP23dm0307101 for NH]. This study was supported in part by Subsidies for Current Expenditures to Private Institutions of Higher Education from the 10.13039/501100012359Promotion and Mutual Aid Corporation for Private Schools of Japan (for MF and NH), through a subaward from 10.13039/501100005731Juntendo University, and from the Research Institute for Diseases of Old Age, Graduate School of Medicine, 10.13039/501100005731Juntendo University (for MF, YH, and NH).

## Data availability statement

The data in this study are available from the corresponding author upon reasonable request.

## CRediT authorship contribution statement

**Mayu Ishiguro:** Writing – review & editing, Writing – original draft, Validation, Investigation, Formal analysis. **Manabu Funayama:** Writing – review & editing, Supervision, Project administration, Methodology, Investigation, Funding acquisition, Formal analysis, Conceptualization. **Taku Hatano:** Writing – review & editing, Writing – original draft, Investigation, Funding acquisition. **Hiroshi Nishida:** Writing – review & editing, Resources. **Yuko Wada:** Writing – review & editing, Resources. **Kazuyuki Noda:** Writing – review & editing, Resources. **Masahiko Tomiyama:** Writing – review & editing, Resources. **Hiroyo Yoshino:** Writing – review & editing, Validation, Methodology, Investigation, Funding acquisition, Data curation. **Yuanzhe Li:** Writing – review & editing, Validatio. **Stephanie Ong:** Writing – review & editing, Validation, Investigation. **Ettore Cioffi:** Writing – review & editing, Validation, Investigation. **Kenya Nishioka:** Writing – review & editing, Investigation. **Nobutaka Hattori:** Writing – review & editing, Supervision, Project administration, Funding acquisition, Conceptualization.

## Declaration of competing interest

The authors declare the following financial interests/personal relationships which may be considered as potential competing interests:Manabu Funayama reports was provided by Promotion and Mutual Aid Corporation for Private Schools of Japan. Nobutaka Hattori reports financial support was provided by 10.13039/501100012359Promotion and Mutual Aid Corporation for Private Schools of Japan. Manabu Funayama reports a relationship with The Michael J Fox Foundation that includes: funding grants. Taku Hatano reports a relationship with Sumitomo Pharma Co Ltd that includes: speaking and lecture fees. Taku Hatano reports a relationship with 10.13039/100008373Takeda Pharmaceutical Company Limited that includes: speaking and lecture fees. Taku Hatano reports a relationship with 10.13039/501100004095Kyowa Kirin Co Ltd that includes: speaking and lecture fees. Taku Hatano reports a relationship with 10.13039/100008792Novartis Pharma Kabushiki Kaisha that includes: speaking and lecture fees. Taku Hatano reports a relationship with 10.13039/501100003769Eisai Co Ltd that includes: speaking and lecture fees. Taku Hatano reports a relationship with 10.13039/100015377Nihon Medi-Physics Co Ltd that includes: speaking and lecture fees. Hiroshi Nishida reports a relationship with Sumitomo Pharma Co Ltd that includes: speaking and lecture fees. Hiroshi Nishida reports a relationship with 10.13039/100008373Takeda Pharmaceutical Company Limited that includes: speaking and lecture fees. Hiroshi Nishida reports a relationship with 10.13039/501100004095Kyowa Kirin Co Ltd that includes: speaking and lecture fees. Hiroshi Nishida reports a relationship with 10.13039/501100007132Otsuka Pharmaceutical Co Ltd that includes: speaking and lecture fees. Hiroshi Nishida reports a relationship with 10.13039/501100003769Eisai Co Ltd that includes: speaking and lecture fees. Hiroshi Nishida reports a relationship with DAIICHI SANKYO COMPANY, LIMITED that includes: speaking and lecture fees. Hiroshi Nishida reports a relationship with 10.13039/501100012351Mitsubishi Tanabe Pharma Corporation that includes: speaking and lecture fees. Hiroshi Nishida reports a relationship with Mochida Pharmaceutical Co Ltd that includes: speaking and lecture fees. Kazuyuki Noda reports a relationship with Sumitomo Pharma Co Ltd that includes: speaking and lecture fees. Kazuyuki Noda reports a relationship with 10.13039/100008373Takeda Pharmaceutical Company Limited that includes: speaking and lecture fees. Kazuyuki Noda reports a relationship with 10.13039/501100007132Otsuka Pharmaceutical Co Ltd that includes: speaking and lecture fees. Kazuyuki Noda reports a relationship with 10.13039/501100004095Kyowa Kirin Co Ltd that includes: speaking and lecture fees. Kazuyuki Noda reports a relationship with 10.13039/100015993Kowa Company Ltd that includes: speaking and lecture fees. Masahiko Tomiyama reports a relationship with Sumitomo Pharma Co Ltd that includes: speaking and lecture fees. Masahiko Tomiyama reports a relationship with 10.13039/100008373Takeda Pharmaceutical Company Limited that includes: speaking and lecture fees. Masahiko Tomiyama reports a relationship with 10.13039/501100004095Kyowa Kirin Co Ltd that includes: speaking and lecture fees. Masahiko Tomiyama reports a relationship with 10.13039/501100013170Ono Pharmaceutical Co Ltd that includes: speaking and lecture fees. Masahiko Tomiyama reports a relationship with 10.13039/501100003769Eisai Co Ltd that includes: speaking and lecture fees. Yuanzhe Li reports a relationship with Eisai Co Ltd that includes: employment. Yuanzhe Li reports a relationship with Nihon Medi-Physics Co Ltd that includes: employment. Nobutaka Hattori reports a relationship with Sumitomo Pharma Co Ltd that includes: board membership, consulting or advisory, paid expert testimony, and speaking and lecture fees. Nobutaka Hattori reports a relationship with 10.13039/100008373Takeda Pharmaceutical Company Limited that includes: board membership, consulting or advisory, paid expert testimony, and speaking and lecture fees. Nobutaka Hattori reports a relationship with 10.13039/501100004095Kyowa Kirin Co Ltd that includes: board membership, consulting or advisory, paid expert testimony, and speaking and lecture fees. Nobutaka Hattori reports a relationship with 10.13039/501100010486Teijin Pharma Limited that includes: board membership, consulting or advisory, paid expert testimony, and speaking and lecture fees. Nobutaka Hattori reports a relationship with PARKINSON Laboratories Co. Ltd that includes: consulting or advisory and equity or stocks. Nobutaka Hattori reports a relationship with 10.13039/501100013170Ono Pharmaceutical Co Ltd that includes: board membership, paid expert testimony, and speaking and lecture fees. Nobutaka Hattori reports a relationship with Riken Center for Brain Science that includes: employment. Nobutaka Hattori reports a relationship with Abbvie 10.13039/100013093GK that includes: speaking and lecture fees. Nobutaka Hattori reports a relationship with 10.13039/501100007132Otsuka Pharmaceutical Co Ltd that includes: speaking and lecture fees. Nobutaka Hattori reports a relationship with 10.13039/100008792Novartis Pharma Kabushiki Kaisha that includes: speaking and lecture fees. Nobutaka Hattori reports a relationship with 10.13039/501100003769Eisai Co Ltd that includes: speaking and lecture fees. Nobutaka Hattori reports a relationship with DAIICHI SANKYO COMPANY, LIMITED that includes: speaking and lecture fees. Nobutaka Hattori reports a relationship with 10.13039/501100013126FP Pharmaceutical Corporation that includes: speaking and lecture fees. Nobutaka Hattori reports a relationship with The Michael J Fox Foundation that includes: funding grants. Nobutaka Hattori reports a relationship with 10.13039/100008440International Parkinson and Movement Disorder Society that includes: funding grants. If there are other authors, they declare that they have no known competing financial interests or personal relationships that could have appeared to influence the work reported in this paper.

## References

[1] Postuma R.B., Berg D., Stern M. (2015). MDS clinical diagnostic criteria for Parkinson's disease. Mov. Disord..

[2] Deng H., Wang P., Jankovic J. (2018). The genetics of Parkinson disease. Ageing Res. Rev..

[3] Nagakubo D., Taira T., Kitaura H. (1997). DJ-1, a novel oncogene which transforms mouse NIH3T3 cells in cooperation with ras. Biochem. Biophys. Res. Commun..

[4] Bonifati V., Rizzu P., van Baren M.J. (2003). Mutations in the DJ-1 gene associated with autosomal recessive early-onset parkinsonism. Science.

[5] Honbou K., Suzuki N.N., Horiuchi M. (2003). The crystal structure of DJ-1, a protein related to male fertility and Parkinson's disease. J. Biol. Chem..

[6] Alcalay R.N., Caccappolo E., Mejia-Santana H. (2010). Frequency of known mutations in early-onset Parkinson disease: implication for genetic counseling: the consortium on risk for early onset Parkinson disease study. Arch. Neurol..

[7] Gibb W.R., Lees A.J. (1988). The relevance of the Lewy body to the pathogenesis of idiopathic Parkinson's disease. J. Neurol. Neurosurg. Psychiatry.

[8] Hayashida A., Li Y., Yoshino H. (2021). The identified clinical features of Parkinson's disease in homo-, heterozygous and digenic variants of PINK1. Neurobiol. Aging.

[9] Untergasser A., Cutcutache I., Koressaar T. (2012). Primer 3--new capabilities and interfaces. Nucleic Acids Res..

[10] Ioannidis N.M., Rothstein J.H., Pejaver V. (2016). REVEL: an Ensemble method for predicting the pathogenicity of rare missense variants. Am. J. Hum. Genet..

[11] Sherry S.T., Ward M., Sirotkin K. (1999). dbSNP-database for single nucleotide polymorphisms and other classes of minor genetic variation. Genome Res..

[12] Hague S., Rogaeva E., Hernandez D. (2003). Early-onset Parkinson's disease caused by a compound heterozygous DJ-1 mutation. Ann. Neurol..

[13] Kasten M., Hartmann C., Hampf J. (2018). Genotype-phenotype relations for the Parkinson's disease genes parkin, PINK1, DJ1: MDSGene systematic review. Mov. Disord..

[14] Guo J.F., Xiao B., Liao B. (2008). Mutation analysis of Parkin, PINK1, DJ-1 and ATP13A2 genes in Chinese patients with autosomal recessive early-onset Parkinsonism. Mov. Disord..

[15] Delva A., Race V., Boon E., Van Laere K., Vandenberghe W. (2021). Parkinson's disease with a homozygous PARK7 mutation and clinical onset at the age of 5 years. Mov Disord Clin Pract.

[16] Bras J.M., Guerreiro R.J., Teo J.T.H. (2014). Atypical parkinsonism-dystonia syndrome caused by a novel DJ1 mutation. Mov Disord Clin Pract.

[17] Abbas M.M., Govindappa S.T., Sudhaman S. (2016). Early Onset Parkinson's disease due to DJ1 mutations: an Indian study. Parkinsonism Relat. Disorders.

[18] Taipa R., Pereira C., Reis I. (2016). DJ-1 linked parkinsonism (PARK7) is associated with Lewy body pathology. Brain.

[19] Stephenson S.E., Djaldetti R., Rafehi H. (2019). Familial early onset Parkinson's disease caused by a homozygous frameshift variant in PARK7: clinical features and literature update. Parkinsonism Relat. Disorders.

[20] Di Nottia M., Masciullo M., Verrigni D. (2017). DJ-1 modulates mitochondrial response to oxidative stress: clues from a novel diagnosis of PARK7. Clin. Genet..

[21] Hanagasi H.A., Giri A., Kartal E. (2016). A novel homozygous DJ1 mutation causes parkinsonism and ALS in a Turkish family. Parkinsonism Relat. Disorders.

[22] Annesi G., Savettieri G., Pugliese P. (2005). DJ-1 mutations and parkinsonism-dementia-amyotrophic lateral sclerosis complex. Ann. Neurol..

[23] Hering R., Strauss K.M., Tao X. (2004). Novel homozygous p.E64D mutation in DJ1 in early onset Parkinson disease (PARK7). Hum. Mutat..

[24] Takanashi M., Funayama M., Matsuura E. (2018). Isolated nigral degeneration without pathological protein aggregation in autopsied brains with LRRK2 p.R1441H homozygous and heterozygous mutations. Acta Neuropathol Commun.

[25] Li Y., Ikeda A., Yoshino H. (2020). Clinical characterization of patients with leucine-rich repeat kinase 2 genetic variants in Japan. J. Hum. Genet..

[26] Ishiguro M., Li Y., Yoshino H. (2021). Clinical manifestations of Parkinson's disease harboring VPS35 retromer complex component p.D620N with long-term follow-up. Parkinsonism Relat. Disorders.

[27] Kiss R., Zhu M., Jojart B. (2017). Structural features of human DJ-1 in distinct Cys 106 oxidative states and their relevance to its loss of function in disease. Biochim. Biophys. Acta Gen. Subj..

[28] Chen J., Li L., Chin L.S. (2010). Parkinson disease protein DJ-1 converts from a zymogen to a protease by carboxyl-terminal cleavage. Hum. Mol. Genet..

